# Dynamic changes in virus-induced volatiles in cotton modulate the orientation and oviposition behavior of the whitefly *Bemisia tabaci*

**DOI:** 10.3389/fphys.2022.1017948

**Published:** 2022-10-10

**Authors:** Suresh M. Nebapure, Karuppan Shankarganesh, Salim Rajna, Kailash Chandra Naga, Dheerendra Pandey, Shubham Gambhir, Koovalamkadu Velayudhan Praveen, Sabtharishi Subramanian

**Affiliations:** ^1^ ICAR-Indian Agricultural Research Institute, New Delhi, India; ^2^ ICAR-Central Institute for Cotton Research, Regional Station, Coimbatore, India; ^3^ ICAR-Central Potato Research Institute, Shimla, India

**Keywords:** whitefly, cotton leaf curl virus, interactions, volatile organic compounds, oviposition, orientation

## Abstract

Manipulation of insect vector behavior by virus-induced plant volatiles is well known. But how the viral disease progression alters the plant volatiles and its effect on vector behavior remains less explored. Our studies tracked changes in volatile profile in progressive infection stages of cotton leaf curl virus (CLCuV) infected plants and their effect on *B. tabaci* behavior. Significant differences in virus titers were noticed between progressive infection stages showing distinct symptoms. Whiteflies initially settled on CLCuV infected plants, but their preference was shifted to healthy plants over time. GC-MS analysis revealed subtle quantitative/qualitative changes in volatile organic compounds (VOCs) between the healthy and selected CLCuV infection stages. VOCs such as hexanal, (E)-2-hexen-1-ol, (+)-α-pinene, (−)-β-pinene, (Z)-3-hexen-1-ol, (+)-sylvestrene, and (1S,2E,6E, 10R)-3,7,11,11-tetramethylbicycloundeca-2,6-diene (Bicyclogermacrene) were associated with the infection stage showing upward curling of leaves; (E)-2-hexen-1-ol, β-myrcene, β-ocimene, and copaene were associated with the infection stage showing downward curling. Validation studies with eight synthetic VOCs indicated that γ-terpinene elicited attraction to *B. tabaci* (Olfactometric Preference Index (OPI) = 1.65), while β-ocimene exhibited strong repellence (OPI = 0.64) and oviposition reduction (66.01%–92.55%). Our studies have demonstrated that progression of CLCuV disease in cotton was associated with dynamic changes in volatile profile which influences the behavioural responses of whitefly, *B.tabaci*. Results have shown that VOCs such as (+)-α-pinene, (−)-β-pinene γ-Terpinene, α-guaiene; 4- hydroxy- 4 methyl-2- pentanone and β-ocimene emitted from Begomovirus infected plants could be the driving force for early attraction and later repellence/oviposition deterrence of *B. tabaci* on virus-infected plants. The findings of this study offer scope for the management of whitefly, *B. tabaci* through semiochemicals.

## Introduction

Insect vectors transmit a majority of plant viruses. Vector–virus interactions are shaped by evolutionary adaptations. These adaptations are complex and dynamic biological processes involving multiple interactions between viruses, insect vectors, and host plants. The cotton whitefly, *Bemisia tabaci* Gennadius (Hemiptera: Aleyrodidae), which is a pest of global importance, transmits over 200 viral diseases in field crops. Among the plant viruses, Begomoviruses are unique, as they are exclusively transmitted by whiteflies. Cotton leaf curl virus (CLCuV) (Begomovirus: Geminiviridae) vectored by the whitefly *B. tabaci* is a serious disease in cotton across Asia and Africa (Sattar et al., 2013). The progression of CLCuV in cotton is associated with changes in virus titer (Shafiq et al., 2017). Different infection stages exhibit distinct symptoms, such as the thickening of veins followed by downward and upward curling and enation or double leaf formation as the disease advances (Idris 1990; Kapur et al., 1994; Brown 2001; Kang et al., 2003).

Although plant palatability to insects is influenced by the nutritive status of the plant (such as sugars and amino acids), volatile organic compounds (VOCs) play a crucial role in manipulating the behavior of insect vectors. Begomoviruses attenuate plant defense responses and cause dynamic changes in the emission of volatiles between healthy and virus-infected plants (Shi et al., 2021). A complex set of virus-vector interactions modulates the behavior of *B. tabaci* (Mauck et al., 2010; Nogia et al., 2014; Jaime et al., 2020) to ensure efficient transmission. Many studies (Fereres et al., 2016; Wang et al., 2017; Shi et al., 2021) have demonstrated the varying effects of volatile cues emitted by virus-infected plants on the behavior of the whitefly *B. tabaci*. Chen et al. (2017) stated that odors dictate *B. tabaci* selection between healthy and Tomato Leaf Curl Virus (TYLCV)-infected plants, wherein virus-infected plants released phenol and 2-ethyl-1-hexanol, leading to the attraction of the vector *B. tabaci* toward infected plants, while Fang et al. (2013) recorded the differential effects of volatiles, namely, β-myrcene, thymine, β-phellandrene, (+)-4-carene, α-humulene and caryophyllene, emitted by Tomato yellow leaf curl virus TYLCV-infected tomato plants on the B and Q biotypes of *B. tabaci*. Li et al. (2014) observed that the βC1 protein of Tomato yellow leaf curl China virus, TYLCCNV, interacted with host plant MYC2 proteins to suppress terpene synthesis, thereby increasing plant preference by its vector, *B. tabaci*.

Oviposition is an important ecological trait associated with virus–vector interactions. The mutualistic relationship between Begomoviruses and *B. tabaci* is reflected by the changes in fitness traits of whiteflies when feeding on virus-infected plants (Li Y. et al., 2014; Chen et al., 2017). While Guo et al., 2010 documented the increased egg laying by *B. tabaci* on virus-infected plants, Nogia et al. (2014) and Mann et al. (2008) observed the opposite.

Several studies have demonstrated the differences in volatile profiles between healthy and virus-infected plants and their possible roles in the behavior of vectors. Nevertheless, there are still many gaps concerning the exact effects of VOCs, as the amplitude of volatile release is influenced by disease dynamics (Claudel et al., 2018). Chang et al. (2021) observed that virus-infected plants, soon after infection, emit volatiles to make them more attractive to insect vectors, thereby promoting virus acquisition. However, after virus acquisition, the resting time of vectors on infected plants is reduced, and the relative preference of the vector is shifted toward uninfected plants (Mauck et al., 2010; Ingwell et al., 2012), probably to facilitate the spread of the virus (Mann et al., 2012; Casteel et al., 2014; Wang et al., 2015). Taking these facts into consideration, we hypothesize that the temporal progression of the virus in the plant causes quantitative and qualitative variations in the release of VOCs, which in turn would differentially modulate the behavior of the vectors. To test our hypothesis, we tracked the virus titers at progressive CLCuV infection stages showing symptoms such as downward and upward curling and enation or double leaf formation in cotton plants and conducted bioassays to compare whiteflies’ settling and oviposition responses on CLCuV-infected cotton plants in these stages. Next, we performed GC–MS analysis to ascertain and identify differentially expressed VOCs associated with specific infection stages. The functional roles of these selected VOCs were further evaluated by using synthetic compounds vis-a-vis whiteflies’ orientation and oviposition responses.

## Materials and methods

### Maintenance of a virus-free *B. tabaci* population

Adult whiteflies (*B. tabaci*) were collected using an aspirator from cotton fields at the ICAR-Indian Agricultural Research Institute (IARI), New Delhi, and reared in insect growth chambers at the Division of Entomology, ICAR-IARI, New Delhi. A homogeneous *B. tabaci* population (raised from a single pair of male and female whiteflies) was maintained on healthy disease-free cotton (Gossypium hirsutum L) plants under optimum rearing conditions (temperature 25 ± 20°C, RH 60%–70%, and a photoperiod of 14:10 (L:D). Diagnostic PCR analysis was performed using a set of primers, 5′CAG​TCG​TTT​GAG​TCC​AGA​CAT3’ (F) and 5′GGTGACAGTTGCATACCATTTG3′(R) (the primers were designed using the sequences of cotton leaf curl Rajasthan virus clone MZ-22, complete genome Accession number. KX656812.1) to ascertain the disease-free nature of healthy cotton plants (Zubair et al., 2017).

### Genetic group status of the *B. tabaci* population

The genetic group status of the *B. tabaci* population was identified by PCR amplification of the mitochondrial cytochrome oxidase 1 gene using the forward primer CI–J–2195 (5′–TTG​ATT​TTT​TGG​TCA​TCC​AGA​AGT–3′) and the reverse primer TL2–N–3014 (5′–TCC​AAT​GCA​CTA​ATC​TGC​CAT​ATT​A–3′) (Dinsdale et al., 2010). Single whiteflies from the population were used in triplicate for DNA extraction using a DNeasy Blood and Tissue kit (Qiagen) following the manufacturer’s protocol. Genetic group identity was determined by direct sequence comparison using the web-based Basic Local Alignment Search Tool algorithm from the NCBI (https://blast.ncbi.nlm.nih.gov/Blast.cgi). Further, phylogenetic analysis was done using Mega 7.0 software, maintaining a well-assigned consensus database of *B. tabaci* genetic groups as the standard (Kanakala and Ghanim, 2019).

### Maintenance of virus-infected plants

Symptomatic diseased cotton plants from the field were confirmed for the presence of the CLCuV virus using diagnostic PCR with the set of primers mentioned above. Whiteflies collected from these plants (viruliferous whiteflies) were used for inoculations. Adult whiteflies (25 no. per plant) were released into cages containing 25-day-old healthy cotton plants and provided an inoculation access period of 24 h. After inoculation, the whiteflies were removed from these plants using a manual aspirator. The temporal progression of CLCuV manifests as distinct symptoms, such as leaves showing upward curling, downward curling (cupping and severe crumpling), double-leaf formation, or enations on the leaf undersides (Sattar et al., 2013) (Supplementary Figure S1). Accordingly, we have chosen three progressive stages of CLCuV infection in cotton plants showing symptoms such as downward and upward curling and enation or double leaf formation for this study. The plants were maintained to express these symptoms, and leaf samples were collected from different infection stages for the preparation of extracts and viral DNA titer estimation. The presence of the cotton leaf curl virus in the infection stages and viruliferous whiteflies was confirmed using the CLCuV-specific gene primers mentioned in the above section.

### Viral DNA quantification for different stages of virus infestation

CLCuV viral titers were estimated by quantifying the copy number of the viral gene through qPCR. DNA isolation was performed using a GeneJET Plant Genomic DNA Purification Kit (Thermo Fisher), and DNA was quantified using a Nanodrop 2000/2000c Spectrophotometer® (Thermo Fisher Scientific, United States). The qPCR mix included 1 µl DNA template (60 ng/μl), 10 μl of Power SYBR Green Master Mix (Applied Biosystems, United Kingdom), 1 μM of each forward and reverse primer (5′TAC​GGC​GAC​TGT​GAA​GAA​TG3’ - Sense and 5′CCT​GTT​CCT​TTG​AAG​CGT​ATT​G3′- Antisense) and nuclease-free H2O to attain a final reaction volume of 20 μl. Two biological replicates and three technical replicates were used to estimate the viral titer for each infection stage. Genomic DNA isolated from a healthy cotton plant was used as a negative control. The qPCR cycling conditions were as follows: 10 min activation at 94°C followed by 40 cycles of 15 s at 94°C and 30 s at 60°C for both annealing and extension. Virus titers were estimated by preparing a standard curve of the plasmid containing coat protein sequences of CLCuV, which was loaded into the same reaction plate with the leaf samples to be quantified for viral loads. The quantification reaction was performed in 96-well optical plates in a Thermocycler (Step One Plus Applied Biosystems real-time PCR, United States). The standard curve was generated by following the method previously presented by Segev et al. (2004). The qPCR amplicon (98 bp) for standard curve generation was cloned into the pTZ57 R/T vector (Thermo Scientific®) following the manufacturer’s protocol. The plasmid was isolated from positive colonies using a Qiagen Plasmid Miniprep kit and linearized by EcoR1 digestion (New England Biolabs). The linearized plasmid was further purified using a Qiagen PCR purification kit. The plasmid concentration was converted to the copy number using a dsDNA copy number calculator (https://cels.uri.edu/gsc/cndna.html), and six serial dilutions (tenfold) of the plasmid were prepared with a starting concentration of 4.54 × 10–6.

### Preparation of leaf extracts

Cotton leaves (100 g each) from healthy and infection stages were extracted with chilled dichloromethane (HPLC grade, CDH®). The chopped leaves were placed into the solvent to maintain a leaf: solvent ratio of 1:3 for 5 h. The extraction bottles were stored in the refrigerator (4°C) to avoid evaporative loss. The extracts were filtered through Whatman No. 1 filter paper and passed through anhydrous Na2SO4 to remove moisture and through activated charcoal for cleanup. The extracts were concentrated under an N2 stream, and the final volume was made up to 5 ml in each sample. The concentrated extracts were stored at −20°C until further use.

### Chemo-profiling of leaf extracts through gas chromatography-mass spectrometry

The leaf extracts from healthy and virus infected plants from progressive infection stages showing upward and downward curling symptoms were passed through a 0.2 µl nylon syringe filter (13 mm) (Omicron Scientific). The filtrate was then used for profiling volatile organic compounds (VOCs) using a Shimadzu QP 2010 Ultra GC–MS (Shimadzu, Kyoto, Japan) equipped with an Rtx-5ms column measuring 30 × 0.25 mm × 0.25 µm. Helium was used as the carrier gas by maintaining a flow rate of 1 ml/min. An autosampler injected 1 μL of each sample with a 5.0 split ratio. The injection temperature was maintained at 230°C. The initial temperature of the oven was 40°C for a 4 min hold time, then it was increased to 220°C at a 5°C/min ramping rate and held for 2 min. Finally, the temperature was increased to 270°C at a ramping rate of 15°C/min. The temperature of the ion source and interface was maintained at 250°C. The compounds were identified by using the inbuilt NIST14 library. The quantitative comparison of compounds was performed using the percent area under the peak for each compound.

### Bioassays

### Settling preference of the whitefly *B. tabaci*

The comparative settling response of the whitefly *B. tabaci* on healthy and CLCuV disease-infected plants was determined by the method described by Mann et al. (2009) with slight modifications. Three healthy and three CLCuV-infected plants showing specific symptoms (upward/downward curling or enation) were placed in an acrylic cage (55 cm × 45 cm × 45 cm). Approximately 150 aviruliferous adult whiteflies were released into the cage equidistant from the plants. The number of whiteflies settled on each plant was recorded at specified intervals, i.e., 2, 6, 12, 24, and 48 h after the release. The mean number of whiteflies settled per plant was calculated. The experiment was replicated three times.

### Orientation response of whiteflies

Plant extracts collected from healthy and CLCuV infection stages were used for assessing the orientation behavior of the whitefly *B. tabaci* using a Y-tube glass olfactometer (Saad et al., 2013) with internal diameter of 25 mm and 13 cm long arms (main, control and source arm) at 60° from each other. The Y-tube was held in vertical position and placed in a blacked out box to avoid visual distractions (Supplementary Figure S2). The stimulus was placed at the top end of source arm and prefiltered, humidified air at 1.5 L/min flow rate was passed through both source and control arm. Pre-starved adult whiteflies (50 per replication) were released into the main arm, and their movements were observed for 20 min. Whiteflies moving farther than 1/3 of the length into the source or control arm were assumed to have made a specific choice. The number of adults that did not move and remained in the main arm was counted as the “no response” category. Five replications were maintained for each extract. The directions of the source arm and control arms were interchanged to avoid directional bias, and the olfactometer was cleaned with alcohol and dried in the oven (100°C) between treatments to remove the influence of residual volatiles, if any, from previous treatments. The experiment was carried out at a temperature of 25 ± 2°C and 55%–60% relative humidity. The preliminary test found that a minimum of 20 µl CLCuV diseased plant extract was required to induce orientation behavior in whiteflies. Accordingly, 20 µl of different extracts was used as a stimulus in the orientation experiments, and DCM served as a control.

In addition to the plant extracts, VOCs showing differential expression were evaluated for orientation response against *B. tabaci*. Eight synthetic VOCs, viz., α-guaiene, γ-terpinene, 4-hydroxy-4 methyl-2-pentanone, (Z)-3-hexen-1-ol, myrcene, (+)-α-pinene, (−)-β-pinene and β-ocimene, were procured from Sigma–Aldrich (United States). Solutions of synthetic compounds were prepared using acetone as a diluent. Ten microlitres of 0.1 percent solution of each synthetic compound were used as a stimulus, and acetone served as a control. The olfactometric preference index was calculated using the formula OPI = 2T/(T + C), where T is the mean attraction rate (%) and C is the mean repulsion rate (%) (Rioja et al., 2018).

### Oviposition responses of whiteflies

The oviposition responses of adult whiteflies to progressive CLCuV infection stages were determined according to Lisha et al. (2003). Freshly emerged, two-day-old adult whiteflies were used for this experiment. Five pairs of adult whiteflies were released into clip cages (2 cm diameter × 2 cm height) containing healthy and CLCuV-infected plants. The mean number of eggs laid per 5 females was recorded 48 h after the release of the whiteflies. Five biological replications were maintained for each treatment.

### Oviposition responses of the whitefly *B. tabaci* to synthetic VOCs

The effects of synthetic volatiles on the oviposition of *B. tabaci* were studied by the clip cage method. Based on the results of range-finding tests (with broad concentrations ranging from 0.01% to 2.0%), the synthetic VOCs were evaluated at six concentrations (0.05%, 0.07%, 0.1%, 0.3%, 0.5%, and 1% v/v) in deionized water containing 0.3% Tween 80. Healthy cotton plants (35 days old) free from virus infestation were sprayed with synthetic VOCs (2 ml) solutions at the respective concentrations using a hand atomizer to ensure uniform coverage on the plants. The leaves were allowed to dry for 1 h, and then ten mated female whiteflies were released into the clip cages containing plants treated with synthetic compounds. Plants treated with deionized water containing 0.3% Tween 80 served as the control. Each concentration was replicated three times on different plants, and three clip cages were used per plant. The mean number of eggs laid per 10 females was recorded 48 h after the release of whiteflies. The oviposition assays were observed under a Leica EZ4 Stereo Zoom microscope with ocular lenses and × 35 magnification (Leica®, United States).

### Statistical analysis

The two-tailed paired *t* test (0.01 and 0.05 level) was used to test for significant differences between the mean numbers of whiteflies settled on the healthy and virus-infected plants at specified time intervals. One-way ANOVA (SPSS 16.0 software) was used to analyze oviposition/orientation responses and to quantify CLCuV viral copy numbers, and Tukey’s test was used to separate the means. The nonparametric Mann–Whitney–Wilcoxon test is considered robust for analyzing a dataset that is not normally distributed. We used this test to ascertain whether the emission of volatile compounds was significantly different between pairs of leaf stages (upward vs. downward, upward vs. healthy, downward vs. healthy). Principal component analysis (PCA) was performed on the emission of volatile compounds using XLSTAT to reveal the compound associations with each other as well as with the infection stages. After PCA, we identified the components that explained the maximum amount of information based on the variance explained.

## Results

### Genetic group status of the whitefly *B. tabaci*

The whitefly population used in this study belonged to the Asia II 1 genetic group of *B. tabaci*, as deduced from phylogenetic analysis and sequence comparison with the consensus database. The representative partial mitochondrial cytochrome oxidase 1 gene sequence of *B. tabaci* used in this study was deposited in the NCBI with accession number MK636818.

### Viral DNA quantification for different stages of virus infestation

**FIGURE 1:**
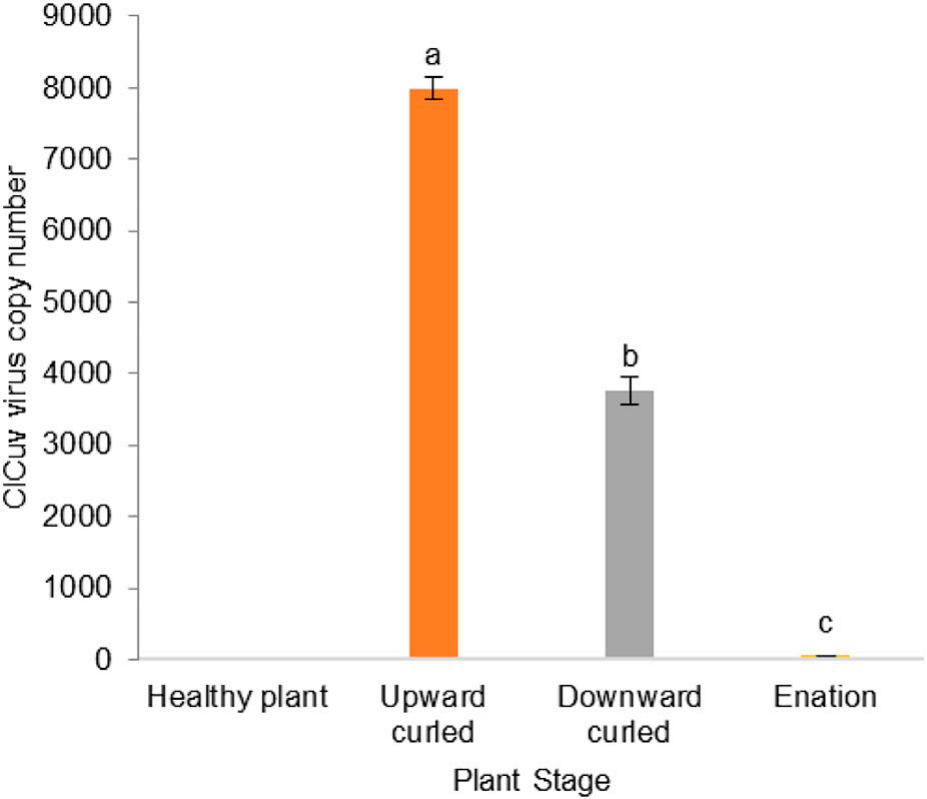
Viral DNA quantification for different stages of CLCuV infected cotton plants.

The incidence of CLCuV was confirmed using diagnostic PCR. The viral titers of CLCuV estimated by qPCR revealed that the healthy plants were free from CLCuV viral copies. Significantly higher CLCuV copies (F (3,8) = 423.01) of the viral gene were observed in the infection stage showing upward-curled leaves (7991.3 ± 151.65/60 ng of DNA) compared to the later stage showing downward-curled leaves (3771.42 ± 336.11/60 ng of DNA). Significantly low viral copy numbers (61.55 ± 3.10/60 ng of DNA) were recorded on leaves showing enation (double leaf) symptoms (Figure 1).

### Settling preference of the whitefly *B. tabaci*

**FIGURE 2:**
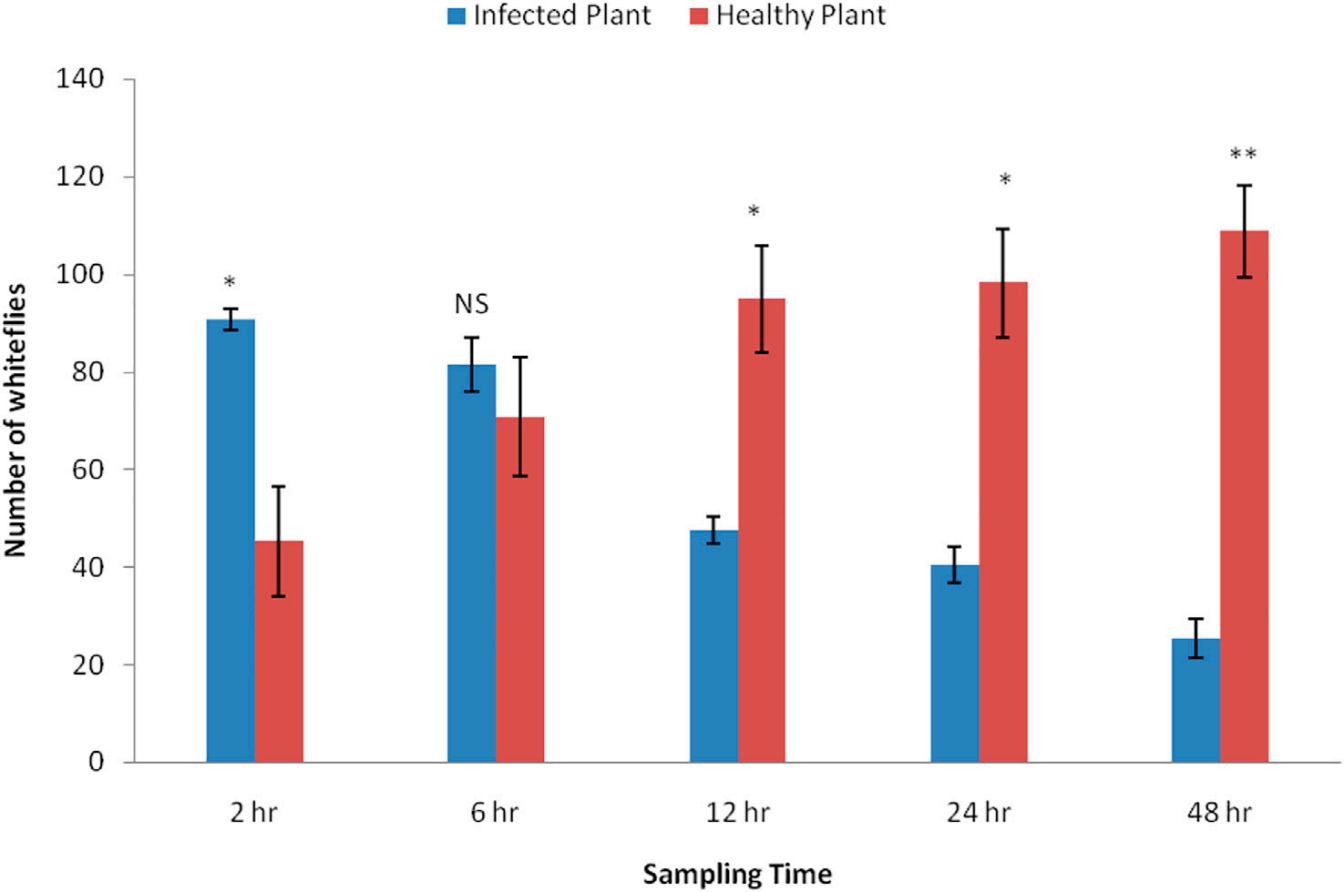
Comparative settling response of whitefly, *Bemisia tabaci* on Healthy Vs. CLCuV infected Cotton plantsThe values indicate the mean number of whiteflies settled per plant by choice method (N = 150). Bars displayed with asterisks denote significant responses (*p < 0.05, **p < 0.01, NS-non significant; two-tailed paired *t*-test).

Significant differences were noticed in the settling responses of adult whiteflies on healthy and CLCuV-infected plants at different time intervals (Figure 2). A significantly higher number of whiteflies settled on virus-infected plants (90.75 ± 11.28) than on healthy plants (45.25 ± 2.02) two hours after release (t = 4.87). No significant difference in settling was observed at 6 h after release (t = 2.88). However, as the time progressed from 12–48 h, a significantly greater number of whiteflies (95–108) preferred to settle on healthy plants (t = 4.45, 4.10, 8.10, respectively for 12, 24, and 48 h) and a significant decline (47–25) was recorded on CLCuV-infected plants.

### Ovipositional responses of the whitefly *B. tabaci*

**FIGURE 3:**
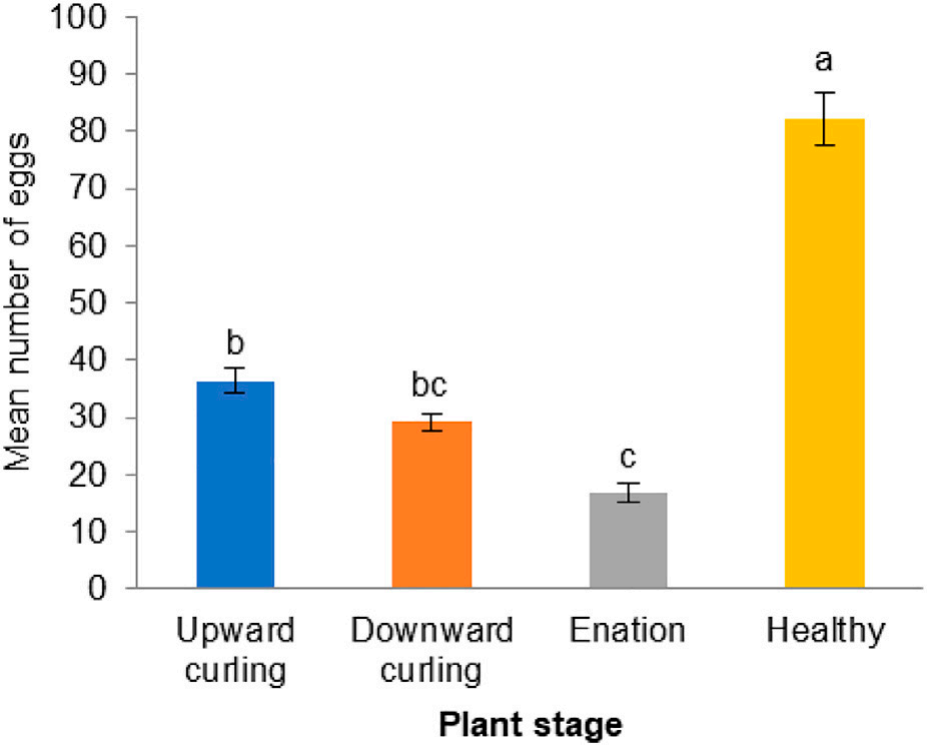
Oviposition responses of Whitefly, *Bemisia tabaci* on healthy and progressive CLCuV infection stages showing upward, downward curling and enation (N = 25) For oviposition response there were five replications each replication consist of one clip cage with five pairs of whiteflies. Bars denoted by common letter indicate a non-significant difference in the mean number of eggs (Tukey’s Test).

The ovipositional preference of the whitefly *B. tabaci* to healthy cotton plants was compared with that on plants in different CLCuV infection stages. A significantly higher number of eggs (82.2 ± 4.49) were laid on the healthy plants than on CLCuV-infected cotton leaves [F (3,16) = 19.934; *p* < 0.00002] (Figure 3). However, whiteflies could not discriminate between CLCuV infection stages showing symptoms of downward curling (29.2 ± 1.48) or upward curling (36.4 ± 2.28). The least number of eggs was deposited on plants showing severe enation symptoms (16.75 ± 1.67).

### Chemo-profiling through GC–MS

GC–MS analysis revealed that healthy plants and plants in two progressive infection stages showing upward and downward curling emitted almost the same set of 23 volatile compounds. However, subtle quantitative differences in certain compounds were noticed between the sets (Table 1). Caryophyllene, (+)-α-pinene, (E)-2-hexenal, 2-hexanol and β-myrcene were the most abundant volatiles identified from healthy, upward-curled, and downward-curled leaves. The volatile release was considered significant between the stages of infection if *p* ≤ 0.05 based on the Mann–Whitney–Wilcoxon test. Accordingly, the release of hexanal, (E)-2-hexen-1-ol, (+)-α-pinene, (−)-β-pinene, (Z)-3-hexen-1-ol, (+)-sylvestrene, and (1S,2E,6E, 10R)-3,7,11,11-tetramethylbicycloundeca-2,6-diene (bicyclogermacrene) was found to be significantly different between the healthy and upward-curled leaves. Similarly, significant differences were noticed between the healthy and downward-curled leaves in the emission of volatiles such as (E)-2-hexen-1-ol, β-myrcene, β-ocimene, and copaene. Between upward- and downward-curled leaves, a significant difference was observed only with respect to 1-methyl-cyclopentanol, (E)-2-hexenal, and (E)-2-hexen-1-ol.

**TABLE 1:** Comparative analysis of volatile organic compounds (VOC’s) from different stages of cotton through GC-MS.

Compound Number	Retentio-n time (Rt) (Min)	Compound	Peak Area (%)			*p*-values from Mann–Whitney–Wilcoxon test				
			Healthy	Upward curled	Downward curled	H*U	H*D	U*D
1	5.488	3-Hexanone	1.39 ± 0.28	1.52 ± 0.02	1.48 ± 0.08	0.513	1.000	0.513
2	5.63	2-Hexanone	1.27 ± 0.25	1.49 ± 0.01	1.4 ± 0.07	0.507	0.827	0.507
3	5.709	1-methyl-Cyclopentanol	0.39 ± 0.09	0.37 ± 0.01	0.43 ± 0.02	0.513	0.827	0.050
4	5.83	3-Hexanol	4.09 ± 0.78	4.02 ± 0.05	4.6 ± 0.26	0.513	0.827	0.127
5	5.912	Hexanal	0.62 ± 0.07	0.85 ± 0.05	0.73 ± 0.09	0.050	0.513	0.127
6	5.995	2-Hexanol	5.1 ± 1.09	5.14 ± 0.08	5.73 ± 0.28	0.827	0.513	0.127
7	7.391	4-hydroxy-4-methyl-2-Pentanone	—	—	1.25 ± 0.23	—	—	—
8	7.688	(E)-2-Hexenal,	6.64 ± 0.93	7.66 ± 0.28	6.19 ± 0.49	0.275	0.513	0.050
9	7.906	(E)-3-Hexen-1-ol,	0.63 ± 0.09	0.55 ± 0.02	0.54 ± 0.05	0.507	0.513	0.825
10	7.891	(E)-2-Hexen-1-ol,	0.57 ± 0	0.13 ± 0	0.45 ± 0.03	0.027	0.083	0.083
11	8.395	Formic acid, hexyl ester	0.2 ± 0.04	0.2 ± 0.01	0.24 ± 0.03	0.507	0.487	0.376
12	10.49	(+)-α.-Pinene	9.31 ± 1.22	13.34 ± 0.34	12.6 ± 0.99	0.050	0.127	0.827
13	12.04	(-)-β-Pinene	1.91 ± 0.22	2.76 ± 0.07	2.54 ± 0.21	0.050	0.127	0.513
14	12.60	β-Myrcene	4.38 ± 0.59	5.92 ± 0.98	6.65 ± 0.64	0.275	0.050	0.275
15	13.21	(Z)-3-Hexen-1-ol	0.17 ± 0.01	0.27 ± 0.04	0.13 ± 0.05	0.050	0.180	0.180
16	13.90	(+)-Sylvestrene	1.05 ± 0.13	1.5 ± 0.05	1.44 ± 0.13	0.050	0.127	0.827
17	14.60	β-Ocimene	1.64 ± 0.21	2.4 ± 0.43	2.75 ± 0.26	0.275	0.050	0.513
18	14.96	γ-Terpinene	—	—	0.37 ± 0.03	—	—	—
19	24.87	Copaene	0.51 ± 0	0.7 ± 0.17	0.4 ± 0.04	0.507	0.046	0.275
20	26.11	Caryophyllene	11 ± 1.65	11.94 ± 0.98	11.28 ± 2.03	0.827	0.827	0.827
21	26.57	α-Guaiene		0.22 ± 0.03	0.28 ± 0.04			0.248
22	27.05	Z,Z,Z-1,5,9,9-tetramethyl-1,4,7,-Cycloundecatriene	3.07 ± 0.39	3.33 ± 0.3	3.18 ± 0.57	0.513	0.827	0.827
23	28.18	(1S,2E,6E,10R)-3,7,11,11 Tetramethyl -bicycloundeca-2,6-diene (Bicyclogermacrene)	0.47 ± 0.1	1.14 ± 0.21	0.85 ± 0.29	0.050	0.275	0.513

Data in the table are mean peak area (±SD) of each compound from three replicates. In each replication extract was obtained from different plant; Identification of the compounds is based on comparison of mass spectra with NIST, inbuilt library; *p*-values from Mann–Whitney–Wilcoxon test to compare emission of volatiles by different stages of infection.

**FIGURE 4:**
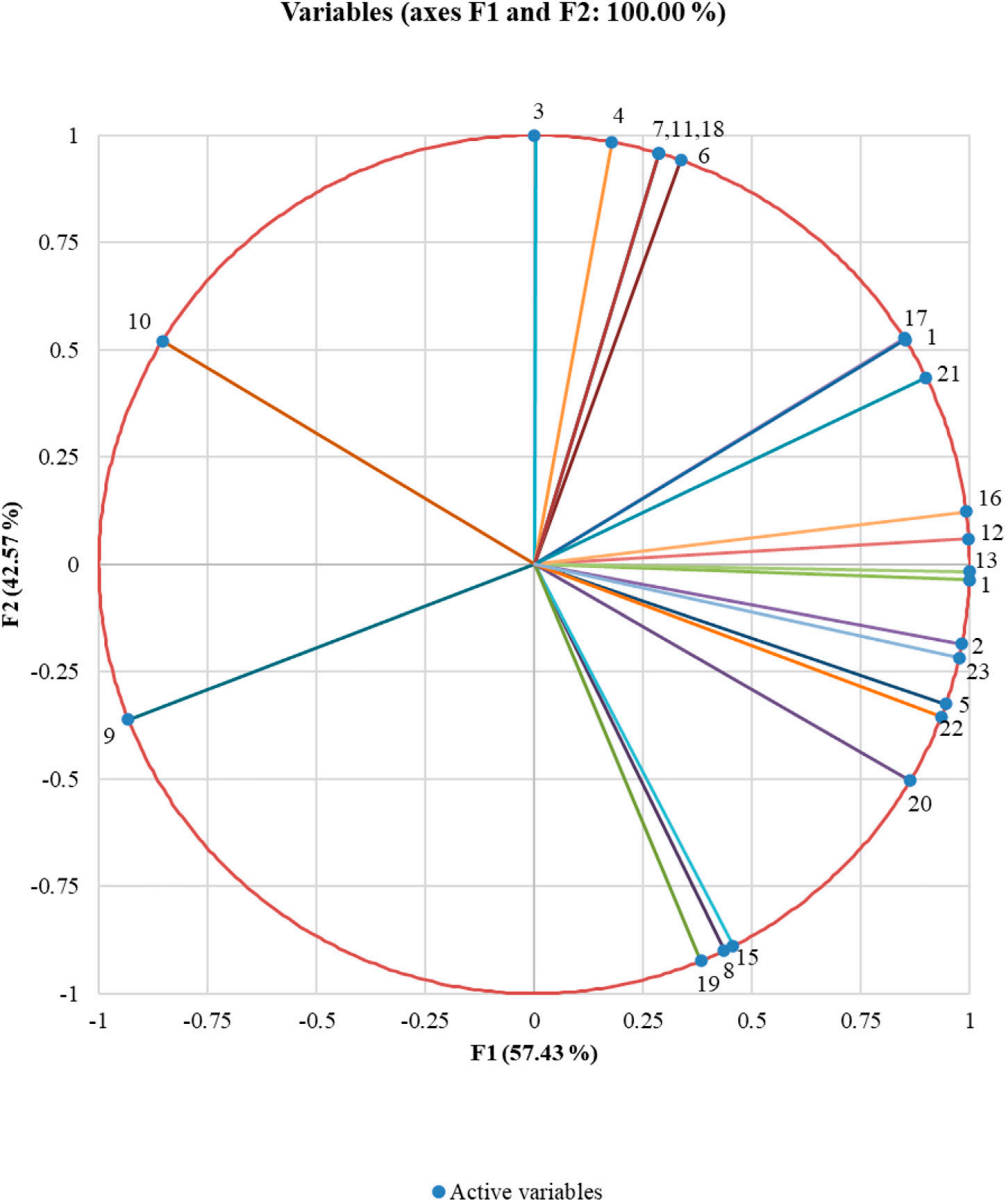
Correlation circle showing the association between volatile compounds. Data points are the mean of three replicates. The closely placed volatile compounds are positively correlated to each other whereas orthogonal places are not correlated; and if they are on the opposite side, then it shows a negative correlation with each other. From the correlation circle, it is clear that all the variables are far away from the centre suggesting the ability of the extracted principal components to explain the variables. It is thus clear that the volatiles (E)-2-hexen-1-ol and caryophyllene are negatively correlated to each other, whereas those closely placed together like (+)-α-pinene and (+)-sylvestrene are positively correlated, and the ones like 1-methyl-cyclopentanol and (−)-β-pinene are not correlated (Supplementary Table S1).

Using PCA, we extracted two principal components that could capture all initial information contained in the variables (23 volatile compounds emitted). While the first PCA explained 57 percent of the total variability, the second could explain the remaining 43 percent. These components are represented by the horizontal axis and the vertical axis, respectively, in the correlation circle (Figure 4). The linkage between the principal components (axis) and the variables (volatiles) can be understood based on the correlation circle and the squared cosines variables table (Supplementary Table S1). 3-Hexanone, 2-hexanone, hexanal, (E)-3-hexen-1-ol, (E)-2-hexen-1-ol, (+)-α-pinene, (−)-β-pinene, β-myrcene, (+)-sylvestrene, β-ocimene, caryophyllene, α-guaiene, Z,Z,Z-1,5,9,9-tetramethyl-1,4,7, cycloundecatriene and (1S,2E,6E, 10R)-3,7,11,11-tetramethylbicycloundeca-2,6-diene (bicyclogermacrene) are the volatiles that are better linked to the horizontal axis representing the first principal component dimension. All others are better linked to the vertical axis representing the second principal component dimension.

**FIGURE 5:**
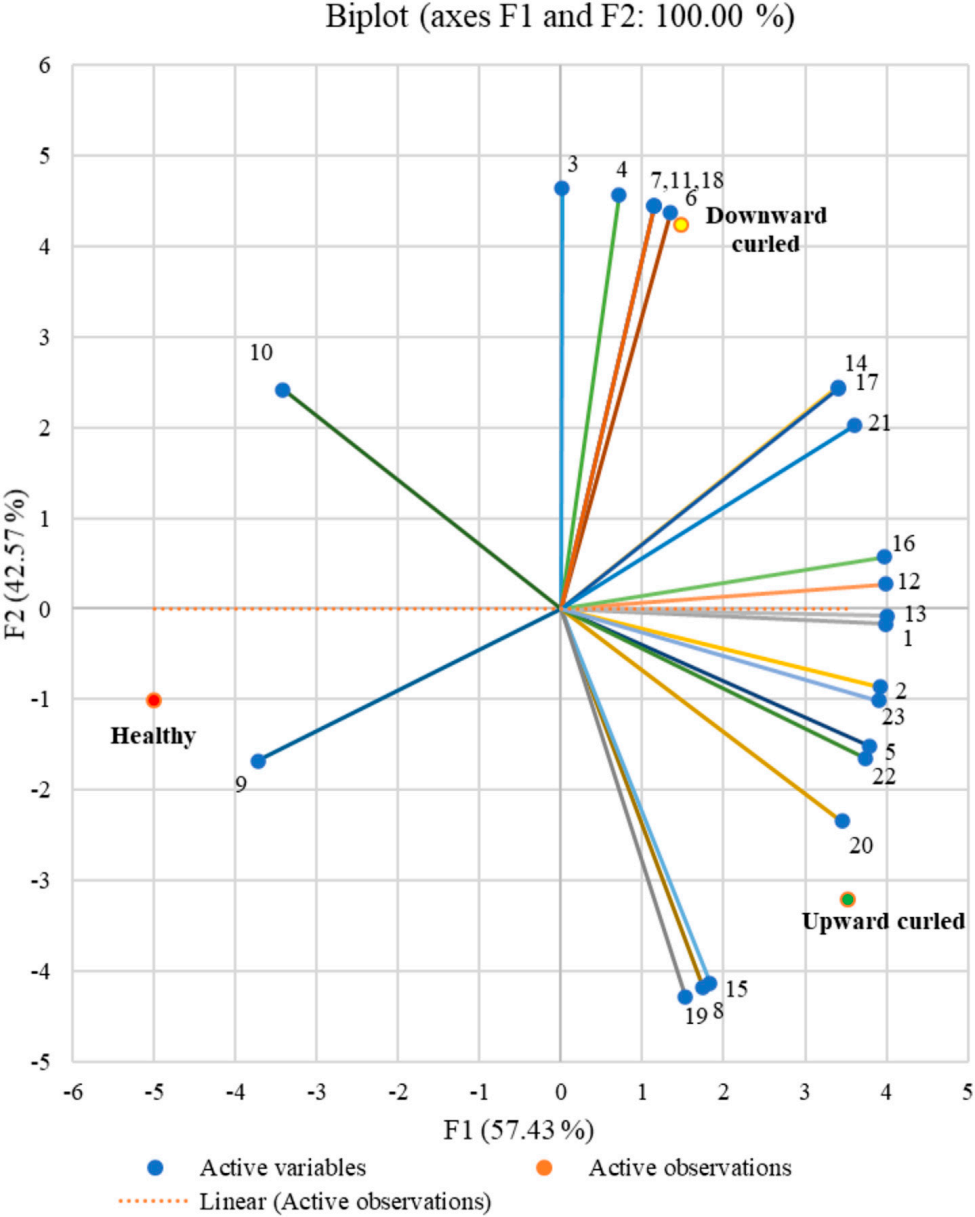
Biplot represents the relationship between different infection stages and volatile emission. The horizontal axis denotes the first PCA dimension representing the upward curled stage, while, the vertical axis denotes the second dimension representing the downward curled stage. Though the observations from healthy stages also contribute to the first PCA, considering a stronger association of the volatile emissions with the upward curled stages, we denote this dimension as upward curled.

Biplot analysis related the different stages (healthy, upward and downward) to the variables (volatile compounds emitted) and to each other. Similar to the correlation circle, the horizontal axis of the biplot represents the first PCA dimension, and the vertical axis represents the second dimension. Each axis can denote different crop stages based on the contribution to the PCA dimensions. The horizontal axis denotes the first PCA dimension representing the upward-curled stage, while the vertical axis denotes the second dimension representing the downward-curled stage. Although the observations from healthy stages also contribute to the first PCA, from Figure 5, it is clear that the volatile emissions are more related to the upward-curled stage than to the healthy stage. For the upward-curled stage, the VOCs 3-hexanone, 2-hexanone, hexanal, (+)-α-pinene, (−)-β-pinene, β-myrcene, (+)-sylvestrene, β-ocimene, caryophyllene, α-guaiene, Z,Z,Z-1,5,9,9-tetramethyl-1,4,7-cycloundecatriene, and (1S,2E,6E, 10R)-3,7,11,11-tetramethylbicycloundeca-2,6-diene (bicyclogermacrene) were all positively correlated, whereas (E)-2-hexen-1-ol and (E)-3-hexen-1-ol were negatively correlated. For the downward-curled stage (E)-2-hexenal, (Z)-3-hexen-1-ol and copaene were negatively correlated, while 1-methyl-cyclopentanol, 3-hexanol, 2-hexanol, 4-hydroxy-4-methyl-2-pentanone, formic acid hexyl ester and γ-terpinene were positively correlated.

Among the VOCs identified as differentially expressed in progressive infection stages of CLCuV, we chose eight compounds, viz., α-guaiene, γ-terpinene, 4-hydroxy-4 methyl-2- pentanone, (Z)-3-hexen-1-ol, myrcene, (+)-α-pinene, (−)-β-pinene and β-ocimene, as candidate VOCs to ascertain their functional roles. These compounds were commercially procured and evaluated against *B. tabaci* in terms of orientation and ovipositional responses.

### Orientation response of the whitefly *B. tabaci*

**FIGURE 6:**
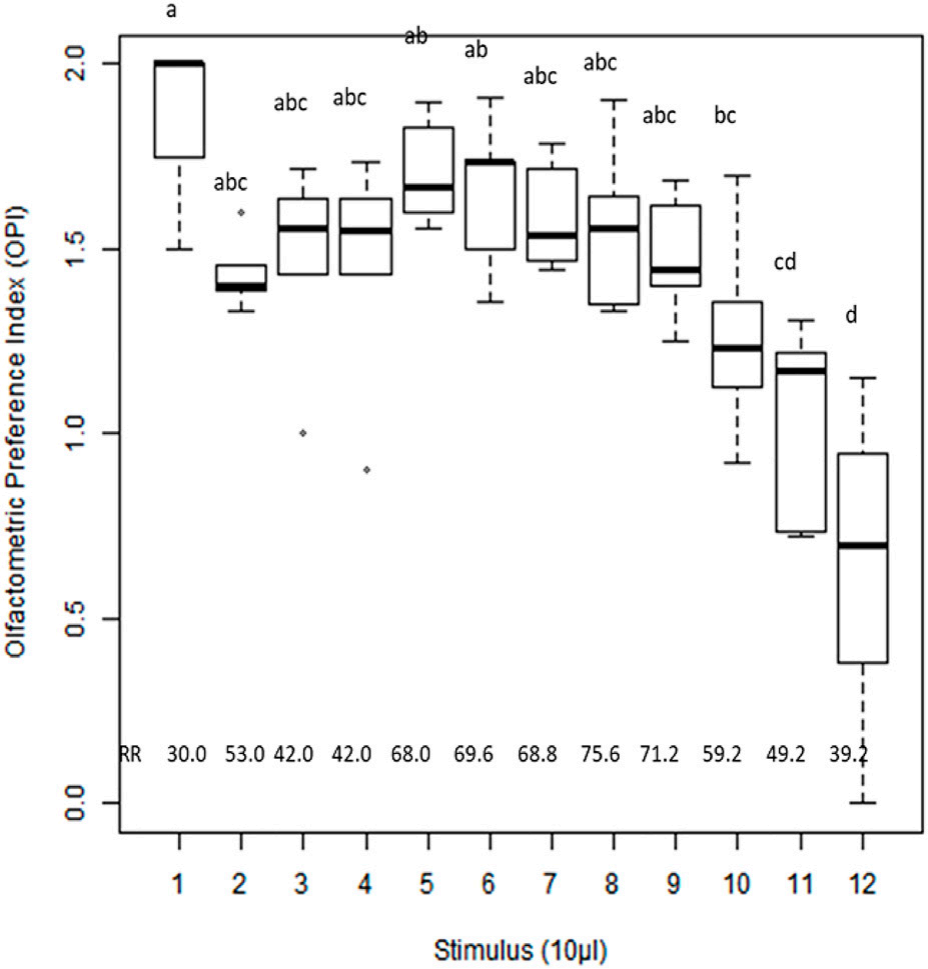
Olfactometric Preference Index (OPI) of whitefly, *Bemisia tabaci* in response to cotton plant extracts of different stages and synthetic plant volatiles. Plant extract (10 µl) or Synthetic compound (10 µl of 0.1% solution) was used as a stimulus. Means with different letters are significantly different (F 9.30; df 11; *p* < 0.00001). OPI values ranged from 0 to 2 (OPI = 1-no olfactometric preference for odor source or control; OPI> 1- preference for treatment odor source arm; OPI<1-preference for control arm). The analysis was done using STAR software. The extract/compound number represent 1:Upward curled leaves extract; 2: Downward curled leaves extract; 3: Enation stage extract; 4:Healthy leaves extract; 5:(+)-α-pinene; 6:γ-Terpinene; 7:α-guaiene; 8:4- Hydroxy- 4 methyl-2- pentanone; 9: (Z)-3-hexen-1-ol; 10:Myrcene; 11: (−)-β-pinene; 12:β-Ocimene.

The orientation responses of whiteflies to extracts of different plant stages (H, UC, DC, and DL) and synthetic volatiles were evaluated using a Y-tube olfactometer, and the olfactometric preference index (OPI) was calculated (Figure 6). Significant differences in OPI values (F = 7.98; df 11; *p* < 0.00001) suggested that the whitefly *B. tabaci* could discriminate between different odor sources. Among the various treatments, the OPI value was significantly low for β-ocimene (0.64), indicating its possible repellent activity toward the whitefly *B. tabaci*. Extracts of upward-curled leaves showed a significantly higher OPI value (1.85), suggesting the positive orientation of the whitefly *B. tabaci*. However, no significant differences in OPI values were observed between the downward curling, enation and healthy leaves. The relative attraction/repulsion responses to different odor sources are presented in Supplementary Figure S3.

### Ovipositional response of *B. tabaci* to synthetic VOCs

**FIGURE 7:**
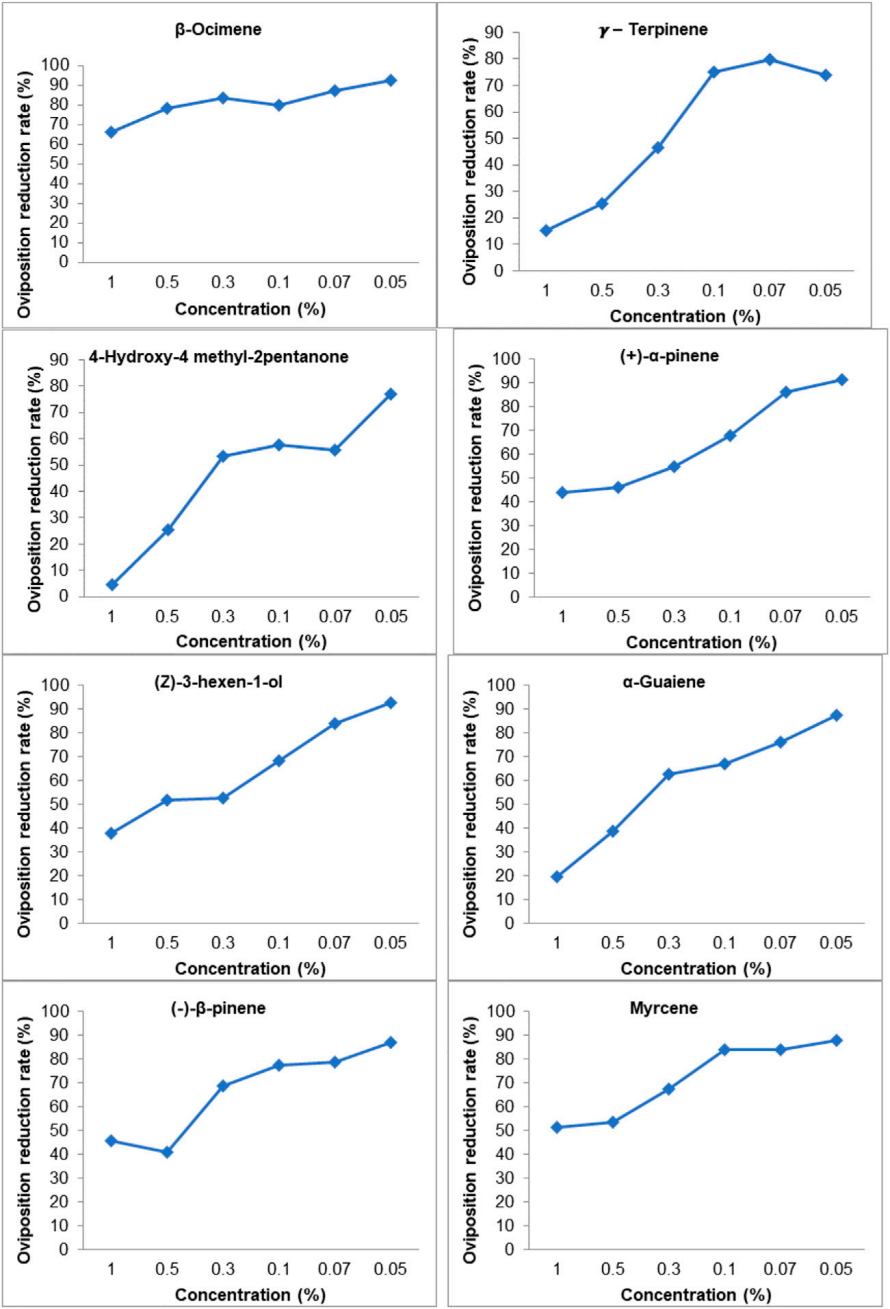
Oviposition reduction rate of *B. tabaci* due to VOC treated cotton leaves, Oviposition reduction rate (%) = [(number eggs on control plant-number eggs on the treated plant)/Number of eggs on control plant] X 100; Values are mean of five replicates.

Selected VOCs showing differential emission rates on GC–MS chemo-profiles of healthy vs. CLCuV infection stages were evaluated for their influence on the ovipositional response of the whitefly *B. tabaci*. A dose-dependent response was noticed for the synthetic VOCs vis-à-vis the egg-laying behavior of *B. tabaci* when synthetic VOCs were tested at incremental concentrations (0.05%–1%). A significant difference in oviposition was observed between the control and VOC treatments (*p* < 0.05; df = 6) (Table 2). *B. tabaci* laid significantly higher numbers of eggs on untreated control plants. Among the treatments, the lowest number of eggs (35.0 ± 8.1 to 7.7 ± 2.3) was recorded on plants treated with β-ocimene, while leaves treated with γ-terpinene received a significantly higher number of eggs. A decrease in the oviposition rate (%) was used to compare the efficacy of commercially available VOCs. Among the compounds tested, β-ocimene showed a 66.01%–92.55% reduction in oviposition at concentrations ranging from 1% to 0.05%. VOCs such as γ-terpinene and 4-hydroxy-4 methyl-2-pentanone, even at a 1.0% concentration, was found to be less effective, with oviposition reduction rates of 15.02%–4.41%, respectively (Figure 7).

**TABLE 2:** Oviposition response of Whitefly *B. tabaci* on cotton plants treated with synthetic volatile organic compounds (VOC’s).

Compound	Mean number of eggs/10 female of *B.tabaci*							*p*-value	F-value
	0.05%	0.07%	0.1%	0.3%	0.5%	1%	Control		
γ—Terpinene	33.3 ± 8.4b	26.0 ± 12.7b	32.0 ± 7.8b	69.0 ± 13.3 ab	96.0 ± 9.1a	109.3 ± 15.6a	128.7 ± 19.1a	0.0002	10.48
Ocimene	7.7 ± 2.3b	13.0 ± 4.2b	20.7 ± 5.2b	17.0 ± 4.0b	22.7 ± 6.4b	35.0 ± 8.1 ab	103.0 ± 36.1a	0.0058	5.08
4-Hydroxy-4 methyl-2- pentanone	26.0 ± 6.4c	50.0 ± 3.5bc	48.0 ± 11.2bc	52.7 ± 2.7bc	84.7 ± 5.2 ab	108.3 ± 5.5a	170.0 ± 15.3a	0.00001	16.26
(+)-α-pinene	9.7 ± 4.1d	16.0 ± 2.5cd	54.5 ± 3.8bcd	51.0 ± 4.4bcd	61.0 ± 3.5bc	63.3 ± 2.0b	113.0 ± 13.4a	0.0001	13.04
(Z)-3-hexen-1-ol	8.0 ± 1.7d	17.0 ± 5.6cd	33.3 ± 4.3bcd	50.0 ± 9.5bc	50.7 ± 6.9bc	65.3 ± 8.6b	105.3 ± 13.2a	0.00001	16.91
α-Guaiene	14.3 ± 3.2d	27.3 ± 2.3d	38.0 ± 5.5d	43.0 ± 5.3cd	70.3 ± 5.2bc	92.0 ± 4.6 ab	114.3 ± 11.2a	0.00001	37.37
(-)-β-pinene	10.7 ± 1.5d	17.3 ± 1.9cd	18.3 ± 2.6cd	25.3 ± 3.8bcd	48.0 ± 3.8b	44.3 ± 4.3bc	81.3 ± 12.8a	0.00001	19.27
Myrcene	13.7 ± 3.8b	18.0 ± 5.7b	18.0 ± 2.1b	36.3 ± 6.7b	51.7 ± 9.0b	54.0 ± 3.2b	110.7 ± 22.4a	0.0001	11.74

Data in the table are the mean number of eggs per 10 females at 48 exposure period by no-choice method; There were three replications, each replication consists of 10 females released in a clip-cage attached on VOC, treated leaf; Values followed by different letters in rows are significantly different at *p* < 0.05 (df = 6) by Tukey’s test.

## Discussion

Virus–vector interactions are thought to be complex and involve multiple interactions between viruses, insect vectors and host plants to enhance transmission. Virus infection alters the morphology and physiology of plants, leading to behavioral changes in insect vectors (Pesti et al., 2019). Werner et al. (2009) established that potato leafroll virus caused changes in the release of volatiles with the temporal progression of the disease in potato plants which had implications on the behaviour of its vector Myzus persicae. Virus–vector interactions are unique in the case of Begomovirus, as these viruses are exclusively transmitted by the whitefly *B. tabaci*. Although Begomovirus–*B. tabaci* interactions mediated by volatiles have widely been reported, how the temporal progression of these viruses influences volatile emissions and their subsequent effect on the behavior of *B. tabaci* largely remain less explored. In this paper, we tracked the changes in the volatile profile in progressive infection stages of CLCuV. We further demonstrated the effects of selected synthetic volatiles on the orientation and ovipositional behavior of *B. tabaci* through validation studies.

The temporal progression of CLCuV is manifested as distinct symptoms (Sattar et al., 2013). We collected the leaves showing symptoms viz., upward curling, downward curling and enation (double leaf) from progressive infection stages of the plants for analysis of virus titer and for estimation of volatiles. Estimation with qPCR revealed a significant rise in virus titers in cotton plants’ early infection stages of CLCuV. A positive correlation between the severity of symptoms and the virus titer has been well documented for Begomovirus infestations (Wege 2007; Ali et al., 2019). We observed that the virus titer was significantly lower or negligible in the advanced stage (enation) of CLCuV infestation. Similar to our results, Shafiq et al. (2017) also observed that the relationship between the symptom severity and the virus titer was not absolute. They observed that plants showing severe symptoms of CLCuV were found to have relatively low virus titers. This is probably because the decline in the viral titer may allow the infected plants to survive for a more extended period to enable the spread of the virus by the vectors (Saunders and Stanley 1999; Briddon et al., 2004).

Settling responses reflect the preferences of insects for particular habitats. Olfactory cues from virus-infected plants are believed to be influencing the settling behavior of the vector *B. tabaci* (Chen et al., 2017). The results of our studies showed that CLCuV-infected plants readily attracted whiteflies, but with the progression of time, the preference of the whiteflies was shifted toward healthy plants (Figure 2). Mann et al. (2009) also observed a similar phenomenon and concluded that a shift in attraction to healthy plants over time facilitated the increased virus spread. The early attraction of the whiteflies to the diseased plants could be driven by a set of favorable volatile cues emanated from virus-infected plants (Fereres et al., 2016; Wang et al., 2017; Shi et al., 2021). Previous studies have established that virus-induced host phenotypes entail an elevated emission of volatile blends normally used by vectors for host location. The attraction behavior imparted due to such volatiles exaggerates the existing host location (Mauck et al., 2010). Our results are consistent with previous reports, and the perceived change in the preference of *B. tabaci* vis-a-vis settling probably enables the spread of the virus to uninfected plants (Mann et al., 2012; Casteel et al., 2014). It is also important to note that vector behaviour could also be manipulated due to physiological changes in infected plants leading to change in nutritional status (sugars, amino acids etc) (Abdullah and Singh, 2004) and accumulation of secondary metabolites (Butter et al., 1992; Ravi et al., 2006). Deteriorating plant quality due to virus infection may also influence the settling of the vector insects (Mauck et al., 2010).

GC–MS profiles were compared between healthy plants and plants in selected CLCuV infection stages showing upward and downward curling of leaves. As this study focused on virus-induced VOCs and their influences on the behavior of whiteflies, we did not include the enation stage in VOC analysis because of its low viral titer. The elevated emission of VOCs from virus-infected plants has been reported by several authors (Pethybridge et al., 2008; Mauck et al., 2014; Fereres et al., 2016; Groen et al., 2016; Liu et al., 2017). Differential emission of volatiles from virus-infected plants modified the behavior of vectors (Claudel et al., 2018). The significant variations observed in the settling responses (Figure 2) of the whiteflies with the temporal progression of CLCuV could be attributed to changes in the emission of volatiles or their blends. It may be probable that the specific set of VOCs emitted during the early stage of viral infection elicited a positive cue, leading to the attraction of *B. tabaci* and enabling the acquisition of the virus. However, after virus acquisition, the changed emission of volatile compounds in the virus-infected plants drove the vectors toward healthy plants and thus promoted the transmission of the virus (Mauck et al., 2010; Ingwell et al., 2012; Dietzgen et al., 2016; Eigenbrode et al., 2018).

There are many reports on the differences in the emission of volatiles between healthy and virus-infected plants. Our investigations specifically explored the possible volatile cues associated with the early attraction and repellence or deterrence of the whitefly *B. tabaci* during later stages of infection as the disease progresses. GC–MS analyses revealed that although both the healthy and CLCuV infection stages emit almost the same set of volatile compounds, subtle quantitative/qualitative changes were noticed in the release of volatiles with the temporal progression of the disease (Table 1). PCA could partition the distinct differences in the volatile profiles between the stages. The extent of the correlations showed a significant affinity between certain VOCs and specific infection stages (Supplementary Table S1; Figure 5). Thus, our studies established the differential expression of volatiles with the temporal progression of CLCuV disease in cotton plants.

Behavioral manipulation of vectors by volatile cues emitted from Begomovirus-infected plants has been well established (Legarrea et al., 2015; Shi et al., 2018). Behavioral assays with a Y-tube olfactometer established that the crude extracts of CLCuV-infected cotton plants elicited more attraction of the whitefly *B. tabaci* than those of healthy cotton plants. Validation studies conducted with select synthetic VOCs also showed differences in the orientation and oviposition behavior of the whitefly *B. tabaci* (Figure 6; Table 2). Our studies demonstrated that (+)-α-pinene, γ-terpinene, α-guaiene, and 4-hydroxy-4 methyl-2-pentanone may be the key attractants emitted Begomovirus-infected plants that attract *B. tabaci*. Significantly higher OPIs for VOCs such as (+)-α-pinene and α-guaiene in upwardly curled leaves may explain the positive orientation of whiteflies at the early stage of CLCuV infection. Incidentally, (Z)-3-hexen-1-ol associated with upward-curled leaves was previously reported as an attractant to the greenhouse whitefly, Trialeurodes vaporariorum (Darshanee et al., 2017).

Among the synthetic VOCs tested, β-ocimene was found to be the least preferred by whiteflies, with an OPI value less than 1. Considering the positive correlation of β-ocimene with the downward-curled stage (Figure 5), the lesser preference of *B. tabaci* for this stage can be attributed to the repellency of β-ocimene to *B. tabaci*. β-ocimene, which is an acyclic monoterpene, has been reported as an herbivore-induced plant repellent cue (Bohlmann et al., 2000). The repellent activity of this compound has previously been demonstrated against *B. tabaci* (Tu and Qin 2017).

Ueda et al. (2019) observed that virus infected plant was less preferred by whitefly, *B. tabaci* for oviposition probably due to accumulation of plant defense substances such as salicylic acid. Further, it was observed that Begomovirus-encoded protein βC1 accounted for the attenuation of plant defense responses (Yang et al., 2008; Zhang et al., 2012). Li et al. (2014) demonstrated that βC1 from Begomovirus interacted with the MYC2 protein, leading to a reduction in the host plant’s terpene synthase (TPS) levels, which in turn enhanced the performance of its vector, B. tabaci. Although Begomovirus-infected plants entice whiteflies with favorable attractant cues, virus-infected plants are not preferred by B. tabaci for egg laying (Mann et al., 2008; Ueda et al., 2019). The results of our studies (Figure 3) also revealed a significantly lower number of eggs on CLCuV-infected plants than on healthy plants. Among the synthetic VOCs, β-ocimene possessed the maximum oviposition inhibition (66.01%–92.55%). Treatment with VOCs such as γ-terpinene and 4-hydroxy-4-methyl-2-pentanone decreased the oviposition rate of B. tabaci by 4%–15%. Earlier studies have shown that Begomovirus infestation alters the fitness of its vector. Fecundity is one of the major fitness traits influencing the buildup of any insect population. Chen et al. (2017) also observed that fecundity of B.tabaci was reduced by feeding on the virus infected plants. However, in contrary to our results, Guo et al. (2010) had reported that feeding on virus-infected plants enhanced the fecundity potential of B. tabaci.

The behavioral response of insects to volatile cues may not strictly adhere to dose-dependent patterns. Interestingly, we observed that certain VOCs inhibited the oviposition of B. tabaci at lower concentrations but not at higher concentrations. Similar to our results, Semmelhack and Wang (2009) observed that although vinegar was an attractant, higher concentrations of the same compound were found to be less attractive to Drosophila melanogaster. Based on attraction or avoidance behavior, the concept of valence is commonly applied to insects. The valence value is considered positive when the insect is attracted and negative when it is repelled. The valence of an odorant often changes with its concentration.

Moreover, the behavioral response of an insect to an odor is dependent not only on the odorant but also on its receptor (Conchou et al., 2019). Many insect odorant receptors respond to more than one odorant, and their binding specificity decreases with increasing odorant concentrations (Andersson et al., 2015). Hence, further studies on the physiology of odorant receptors may shed light on the valence of certain VOCs relative to the ovipositional behavior of B. tabaci.

**FIGURE 8:**
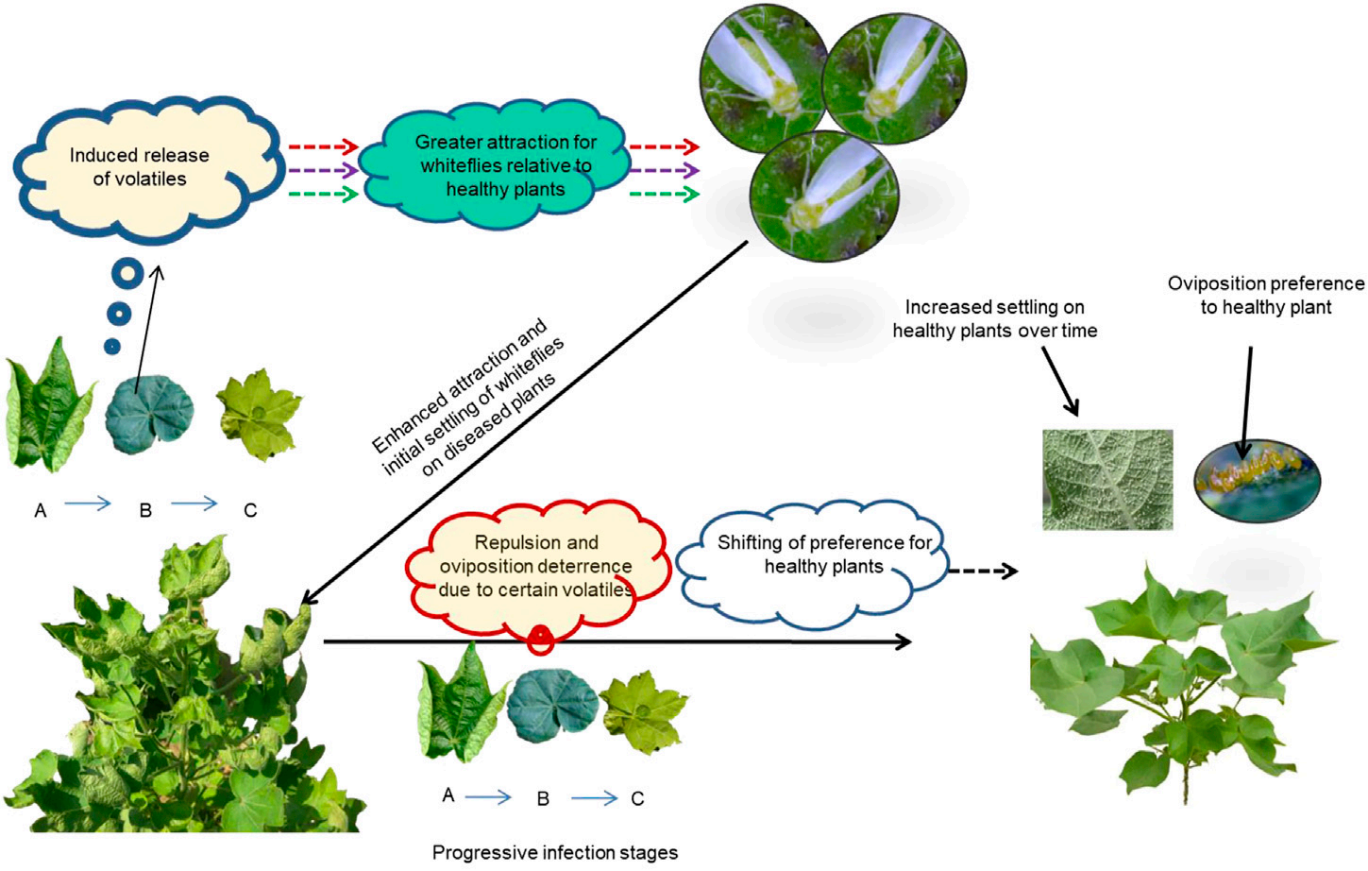
Dynamic interactions between Cotton Leaf Curl Virus and its vector, *Bemisia tabaci*. Although healthy cotton plants are attractive to *B. tabaci* utilizing a natural blend of volatile organic compounds (VOCs), qualitative and quantitative changes occur in the release of VOCs from CLCuV infection stages with the progression of the disease as shown by symptoms **(A)** Upward curling, **(B)** Downward curling and **(C)** Enation/double leaf. The dynamic changes in the release of certain VOCs from diseased plants exaggerated the attraction cues resulting in higher settling of whiteflies on the diseased plant in the initial phase but over time, whiteflies preferred healthy plants for settling and oviposition, driven by repulsive VOCs from diseased plants.

Based on the association of VOCs with the specific infection stages and behavioral responses of B. tabaci to different VOCs, we can infer that early attraction to virus-infected plants can probably be attributed to the emission of compounds such as (+)-α-pinene, γ-terpinene, α-guaiene and 4-hydroxy-4 methyl-2-pentanone. The increased release of repellents such as β-ocimene in later stages of virus infestation can be associated with the shift in the preference of whiteflies toward healthy plants. Thus, the temporal progression of virus infestation causes dynamic changes in the release of volatiles in host plants, and these compounds differentially modulate the behavior of whiteflies, as depicted in the thematic illustration in Figure 8.

The VOCs elucidated in our study are only indicative correlating with disease progression, the release of compounds may vary with crop varieties. Moreover, multiple stages of disease progression may present in field conditions. Several studies have also pointed out that appropriate blends or combinations of VOCs elicit stronger behavioral responses than single compounds (Bruce and Pickett, 2011). Hence, further studies are needed to elucidate the possible role of Begomovirus-induced volatiles and their blends in the behavioral manipulation of *B. tabaci*.

## Conclusion

Begomoviruses are exclusively transmitted by the whitefly *B. tabaci*, and a coevolutionary relationship exists between the virus and the vector. Begomovirus (e.g., CLCuV) modulates the behavior of whiteflies in such a way that the vectors are attracted toward virus-infected plants, and after the acquisition of the virus, the preference is shifted toward healthy plants, thereby ensuring the effective transmission of the virus. Our studies provided empirical evidence of dynamic changes in the volatile profile in cotton plants with the temporal progression of CLCuV. The influence of differentially induced volatiles on the orientation and oviposition behavior of *B. tabaci* was validated by using synthetic VOCs. This differential attraction/repulsion/oviposition deterrence of whiteflies by Begomovirus-infected plants could be manipulated through strategies such as push–pull or repellent and oviposition deterrents or lures to minimize the damage caused by the whitefly *B. tabaci* and its vectoral ability. The leads obtained from this study provide a scope for the management of the whitefly *B. tabaci* through semiochemicals.

## Data Availability

The datasets presented in this study can be found in online repositories. The names of the repository/repositories and accession number(s) can be found in the article/Supplementary Material.
