# Supplementary material for: Dynamic changes in virus-induced volatiles in cotton modulate the orientation and oviposition behavior of the whitefly Bemisia tabaci
Source: Front Physiol. 2022 Oct 10;13:1017948. doi: 10.3389/fphys.2022.1017948 (PMC9589893; doi:10.3389/fphys.2022.1017948)
Supplement: Supplementary file 1 [file Table1.docx]

**Supplementary Table 1** Squared cosines of the variables showing association with principal components

| **Compound Number** | **Volatile compound emitted** | **F1**  **(First PC)** | **F2**  **(Second PC)** |
| --- | --- | --- | --- |
| 1 | 3-Hexanone | **0.999** | 0.001 |
| 2 | 2-Hexanone | **0.966** | 0.034 |
| 3 | 1-methyl-Cyclopentanol | 0.000 | **1.000** |
| 4 | 3-Hexanol | 0.032 | **0.968** |
| 5 | Hexanal | **0.894** | 0.106 |
| 6 | 2-Hexanol | 0.113 | **0.887** |
| 7 | 4-hydroxy-4-methyl-2-Pentanone | 0.082 | **0.918** |
| 8 | (E)-2-Hexenal, | 0.191 | **0.809** |
| 9 | (E)-3-Hexen-1-ol, | **0.869** | 0.131 |
| 10 | (E)-2-Hexen-1-ol, | **0.729** | 0.271 |
| 11 | Formic acid, hexyl ester | 0.082 | **0.918** |
| 12 | (+)-α.-Pinene | **0.997** | 0.003 |
| 13 | (-)-β-Pinene | **1.000** | 0.000 |
| 14 | β-Myrcene | **0.722** | 0.278 |
| 15 | (Z)-3-Hexen-1-ol | 0.209 | **0.791** |
| 16 | (+)-Sylvestrene | **0.985** | 0.015 |
| 17 | β-Ocimene | **0.726** | 0.274 |
| 18 | 𝜸-Terpinene | 0.082 | **0.918** |
| 19 | Copaene | 0.147 | **0.853** |
| 20 | Caryophyllene | **0.746** | 0.254 |
| 21 | α-Guaiene | **0.811** | 0.189 |
| 22 | Z,Z,Z-1,5,9,9-tetramethyl-1,4,7,-Cycloundecatriene | **0.874** | 0.126 |
| 23 | (1S,2E,6E,10R)-3,7,11,11 Tetramethyl -bicycloundeca-2,6-diene (Bicyclogermacrene) | **0.952** | 0.048 |

Values represented in bold correspond for each variable to the factor for which the squared cosine is the largest
