# Supplementary material for: Dynamic changes in virus-induced volatiles in cotton modulate the orientation and oviposition behavior of the whitefly Bemisia tabaci
Source: Front Physiol. 2022 Oct 10;13:1017948. doi: 10.3389/fphys.2022.1017948 (PMC9589893; doi:10.3389/fphys.2022.1017948)
Supplement: Supplementary file 2 [file Image1.pdf]

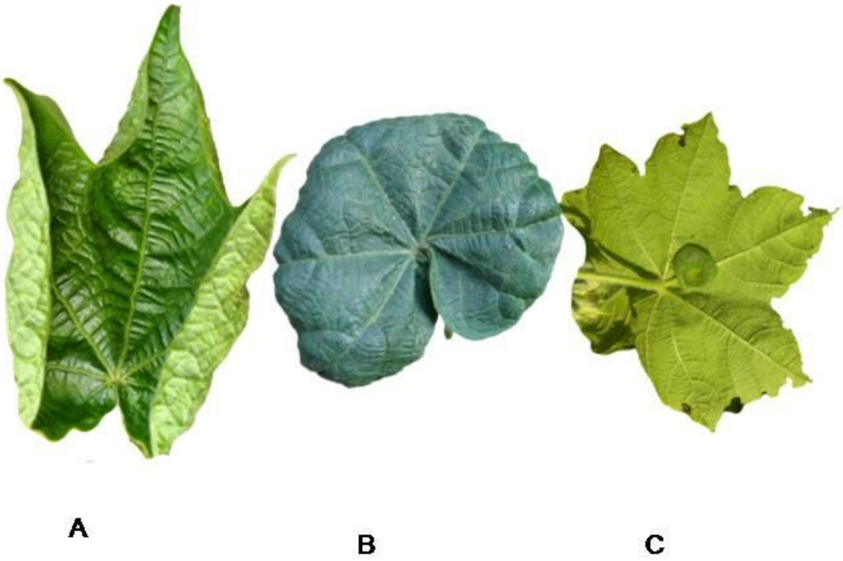

Supplementary fig. 1 Progressive infection stages of CLCuV disease in cotton plant showing distinct symptoms :

A) Upward curling, B) Downward curling, C) Enation or double leaf

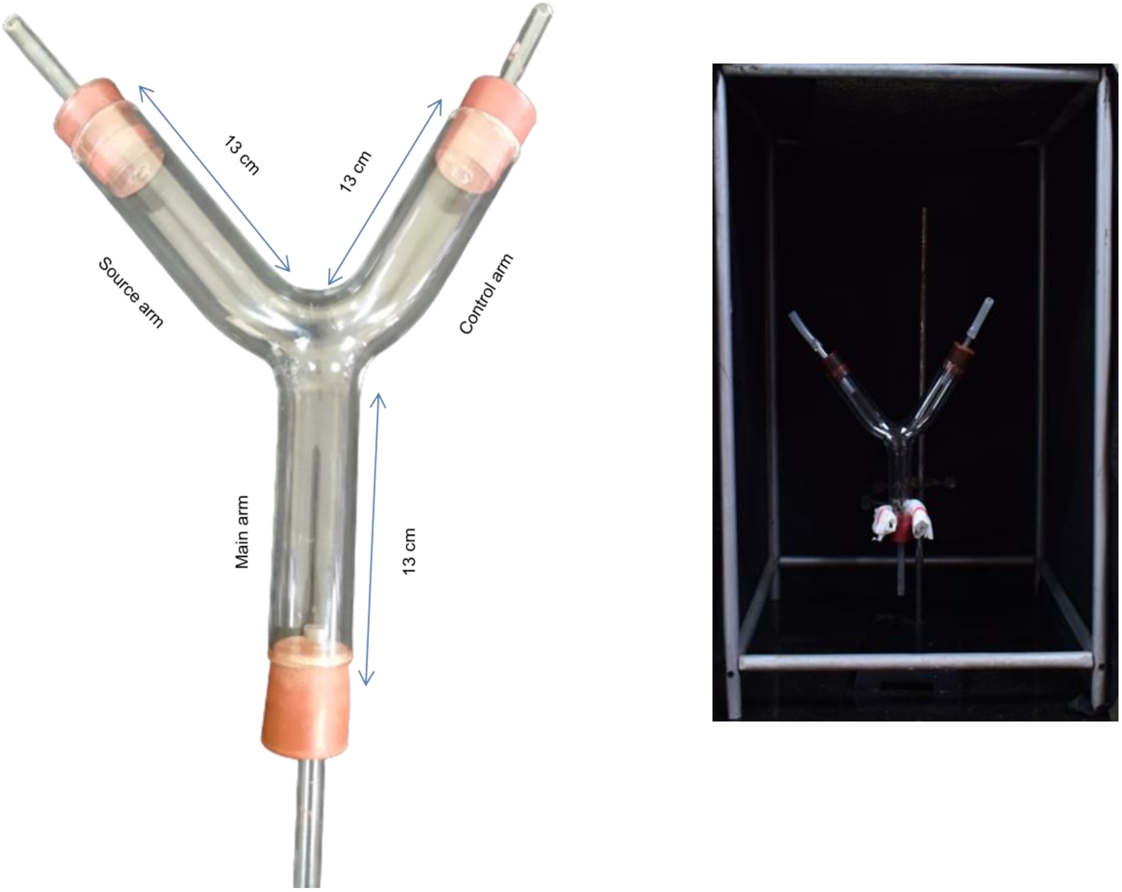

A

B

Supplementary fig. 2

A : Y-tube glass olfactometer used in orientation studies of whitefly, *Bemisia tabaci* ;

B: Y tube Olfactometer set up

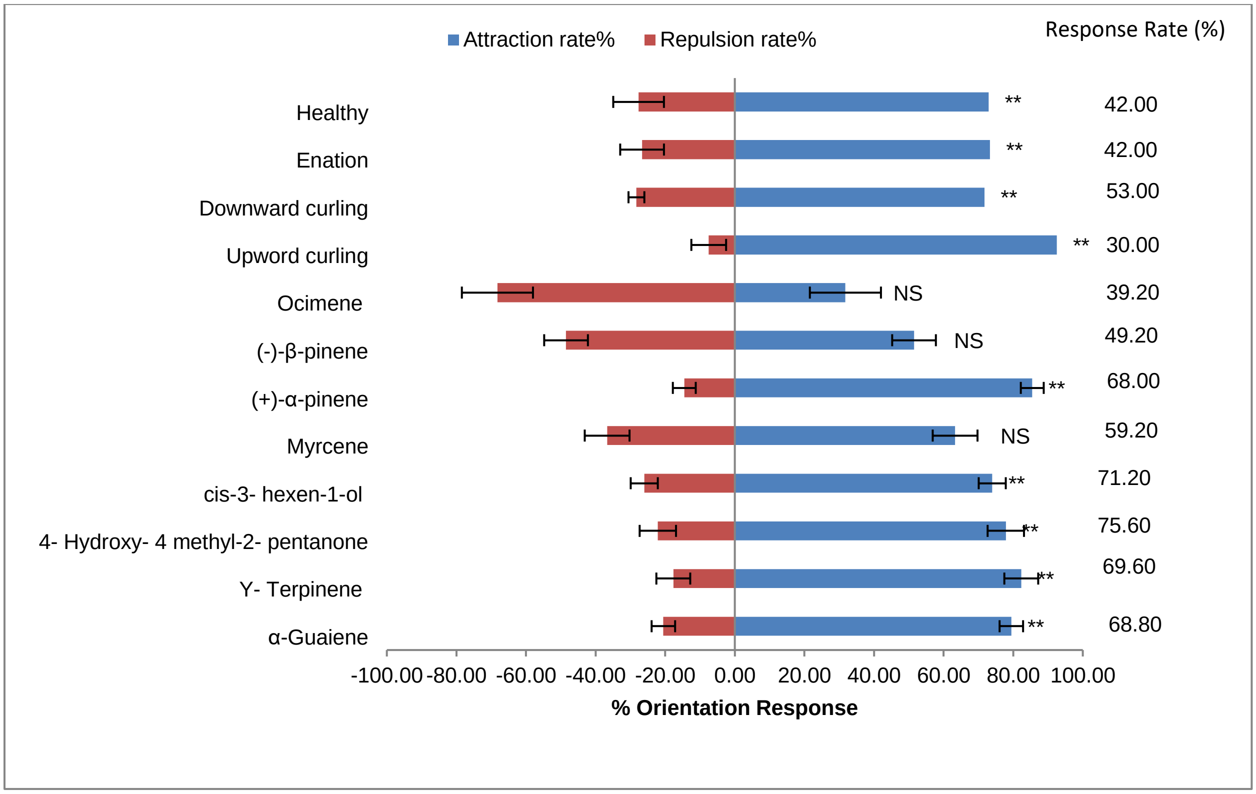

Supplementary fig. 3 Orientation responses of whitefly, *Bemisia tabaci* (N= 250) to odour sources (Healthy and different CLCuV infected stages of cotton plant and synthetic VOC's) through Y-tube olfactometer. Data presented is attraction rate (%) (Attraction Rate (%)=(Insect showing taxis/Responding insects) X 100, Repulsion Rate (%) (Repulsion Rate (%)=[(Responding insects-Insect showing taxis)/Responding Insects] X 100 and Response rate (%) (Response Rate (%)= (Responding Insects/Total number of insects tested) X 100; Error bars represents standard error of mean percent response. Bars denoted by asterisks indicate a significant response for the VOC (* $p<0.05$, ** $P< 0.01$, NS-non significant; two-tailed paired t-test).
